# Using Multi-Scale Genetic, Neuroimaging and Clinical Data for Predicting Alzheimer’s Disease and Reconstruction of Relevant Biological Mechanisms

**DOI:** 10.1038/s41598-018-29433-3

**Published:** 2018-07-24

**Authors:** Shashank Khanna, Daniel Domingo-Fernández, Anandhi Iyappan, Mohammad Asif Emon, Martin Hofmann-Apitius, Holger Fröhlich

**Affiliations:** 1 Department of Bioinformatics, Fraunhofer Institute for Algorithms and Scientific Computing (SCAI), Schloss Birlinghoven, Sankt Augustin, 53754 Germany; 2 Bonn-Aachen International Center for Information Technology (B-IT), University of Bonn, 53113 Bonn, Germany; 3 UCB Biosciences GmbH, Alfred-Nobel Str. 10, 40789 Monheim, Germany

## Abstract

Alzheimer’s Disease (AD) is among the most frequent neuro-degenerative diseases. Early diagnosis is essential for successful disease management and chance to attenuate symptoms by disease modifying drugs. In the past, a number of cerebrospinal fluid (CSF), plasma and neuro-imaging based biomarkers have been proposed. Still, in current clinical practice, AD diagnosis cannot be made until the patient shows clear signs of cognitive decline, which can partially be attributed to the multi-factorial nature of AD. In this work, we integrated genotype information, neuro-imaging as well as clinical data (including neuro-psychological measures) from ~900 normal and mild cognitively impaired (MCI) individuals and developed a highly accurate machine learning model to predict the time until AD is diagnosed. We performed an in-depth investigation of the relevant baseline characteristics that contributed to the AD risk prediction. More specifically, we used Bayesian Networks to uncover the interplay across biological scales between neuro-psychological assessment scores, single genetic variants, pathways and neuro-imaging related features. Together with information extracted from the literature, this allowed us to partially reconstruct biological mechanisms that could play a role in the conversion of normal/MCI into AD pathology. This in turn may open the door to novel therapeutic options in the future.

## Introduction

Alzheimer’s Disease (AD) is among the most frequent neuro-degenerative diseases in people above 65 and affects more than 45 Million people worldwide^1^. It is a chronic disease that usually starts slowly with a pre-symptomatic phase and worsens over time^2^. Early diagnosis is essential for successful disease management and chance to attenuate symptoms by disease modifying drugs. In the past, a number of cerebrospinal fluid (CSF), plasma and neuro-imaging based biomarkers have been proposed for that purpose^3^. Still, in current clinical practice, AD diagnosis cannot be made until the patient shows clear signs of cognitive decline, which can partially be attributed to the multi-factorial nature of AD^4^. AD pathology covers multiple biological scales, ranging from disease risk increasing genomic variants over altered intra-cellular signaling events and regional brain atrophy up to neuro-psychological behavior^5^. Hence, there is a need for establishing robust biomarker signatures covering multiple biological scales, which allow for early AD diagnosis. Several authors proposed models, which discriminate between AD and mild cognitively impaired (MCI) patients using subsets of markers from different data modalities^5–9^. A model to predict the time to conversion from 346 MCI into AD based on clinical data, neuro-imaging features and highly restricted genotype information (only 2 SNPs) was developed by Lee *et al*.^10^. The authors developed a Bayesian functional linear Cox model, which they evaluated based on simulation studies. Based on these simulations they reported an integrated area under ROC curve of 84%. In addition to the general limitation of such a purely simulation based validation the actually utility for clinical practice would have to be validated in a follow-up study first. Moreover, the biological mechanisms driving the MCI to AD conversion remain entirely unclear.

More recently, Li *et al*.^11^ individually investigated different baseline cognitive, neuro-psychological and neuro-imaging scores to predict MCI to AD conversion. The authors employed univariate Cox models with covariate adjustments for age, gender, APOE4 status and education level. Using 6-fold cross-validation they reported a time dependent area under ROC curves of 68–81% for the respective scores. Once again, the biological mechanisms driving the MCI to AD conversion remain unclear.

The goal of this work was two-fold: First, our aim was to establish a multivariate, multi-modal predictive model for the time to AD conversion of normal/MCI patients and to identify most relevant prognostic features. Our model integrated rich genotype information (including newly developed SNP functional pathway impact scores), neuro-imaging (volume measurements of brain regions, PET scan results) as well as clinical data from 900 normal and MCI individuals extracted from the Alzheimer’ s Disease Neuroimaging Initiative (ADNI) (http://adni.loni.usc.edu/), a large scale observational study started in 2004 to evaluate the use of diverse types of biomarkers in clinical practice. A second aim of this work was to better understand the biological mechanisms driving the conversion of normal/MCI into AD pathology, which may ultimately open the door to novel therapeutic options. To this end, we employed a combination of data driven probabilistic and knowledge driven mechanistic approaches. More specifically, we used Bayesian Networks to uncover the interplay across biological scales between genetic variants, pathways, PET scan results and neuro-imaging related features. Together with manually curated cause-effect chains extracted from the literature, this allowed us to partially reconstruct biological mechanisms that could play a role in the conversion of normal/MCI into AD pathology.

## Results

### Overview about Approach

We extracted multi-modal baseline data from 315 normal and 609 mild cognitively impaired (MCI) patients from the ADNI database. 14 (4.4%) of the normal and 238 (39%) of the MCI patients developed AD during the 96 months of the study. We cannot exclude that patients without transition to AD pathology during study time developed AD later. Hence, their disease outcome has to be viewed as right censored.

**Figure 1:**
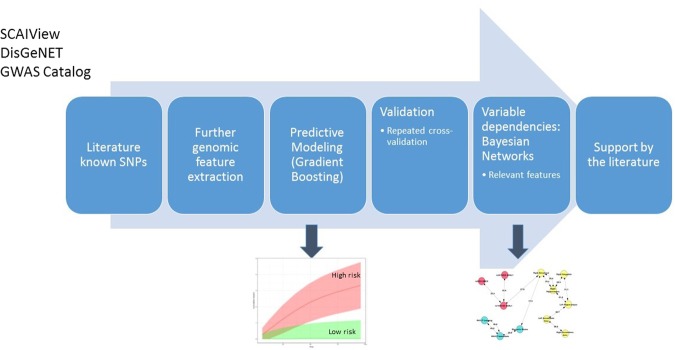
Overall approach to analyze ADNI data.

The clinical baseline data of the altogether 924 patients used in this work comprised 73 variables with diagnosis, demographic information, age, gender, education level, neuro-psychological test, MRI and PET scan results, volume measurements of different brain regions as well as 300,000 single nucleotide polymorphisms (SNPs), which are commonly available from both ADNI1 and ADNI2/GO studies. Our overall approach to analyze these data and reduce their complexity contained six steps that are outlined in Fig. 1:

1. Literature mining of known disease associated SNPs (see Methods for details).
2. Further genomic feature extraction based on global population structure (principal components) plus a newly developed score to measure putative pathway impact of SNPs on individual patient level.
3. Development and evaluation of a predictive time-to-event model for normal/MCI to AD transition.
4. Estimation of (partially causal) dependencies between relevant features in the predictive model via Bayesian Network (BN) structure learning.
5. Validation of the BN with literature derived cause-effect relationships.

Steps 1. and 2. resulted into 313 pathway impact scores, 363 SNPs and 32 principal components that were added to the above mentioned 73 clinical baseline variables.

### Predicting the Time Dependent Alzheimer’s Disease Risk and Identification of Associated Relevant Baseline Characteristics

#### AD Can Be Predicted Accurately

We developed an approach to appropriately integrate the multi-modal data used in this work within a machine learning framework to predict the AD risk for MCI and pre-symptomatic patients (see Methods for details). Our approach uses a weighted ensemble of constraint decision trees - a Gradient Boosting Machine (GBM^12^) - to combine most relevant pathways, SNPs, principal components and clinical baseline variables into a final patient specific prediction score. GBM is an established machine learning method that is – due to its nature as an ensemble of decision trees – well suited to integrate heterogeneous data types (e.g. clinical features plus SNPs) on rather different numerical scales, as in our application^13^. Moreover, GBM allow for an embedded subset selection of most relevant features and appropriately deal with missing values in clinical data.

The prediction performance of our developed GBM based algorithm was assessed via a 10 times repeated 10-fold cross-validation procedure: Cross-validation randomly splits the overall data into *k* (here 10) folds, while successively one of these folds is left out for model validation and the rest for model training. The ability to predict the time to first AD diagnosis for patients in the validation set of each of the 10 GBM models was assessed via Harrell’s concordance/C-index^14^, which is a generalization of the area under ROC curve measure used in binary classification. The C-index ranges from 0 to 1, where 0.5 indicates chance level.

**Figure 2:**
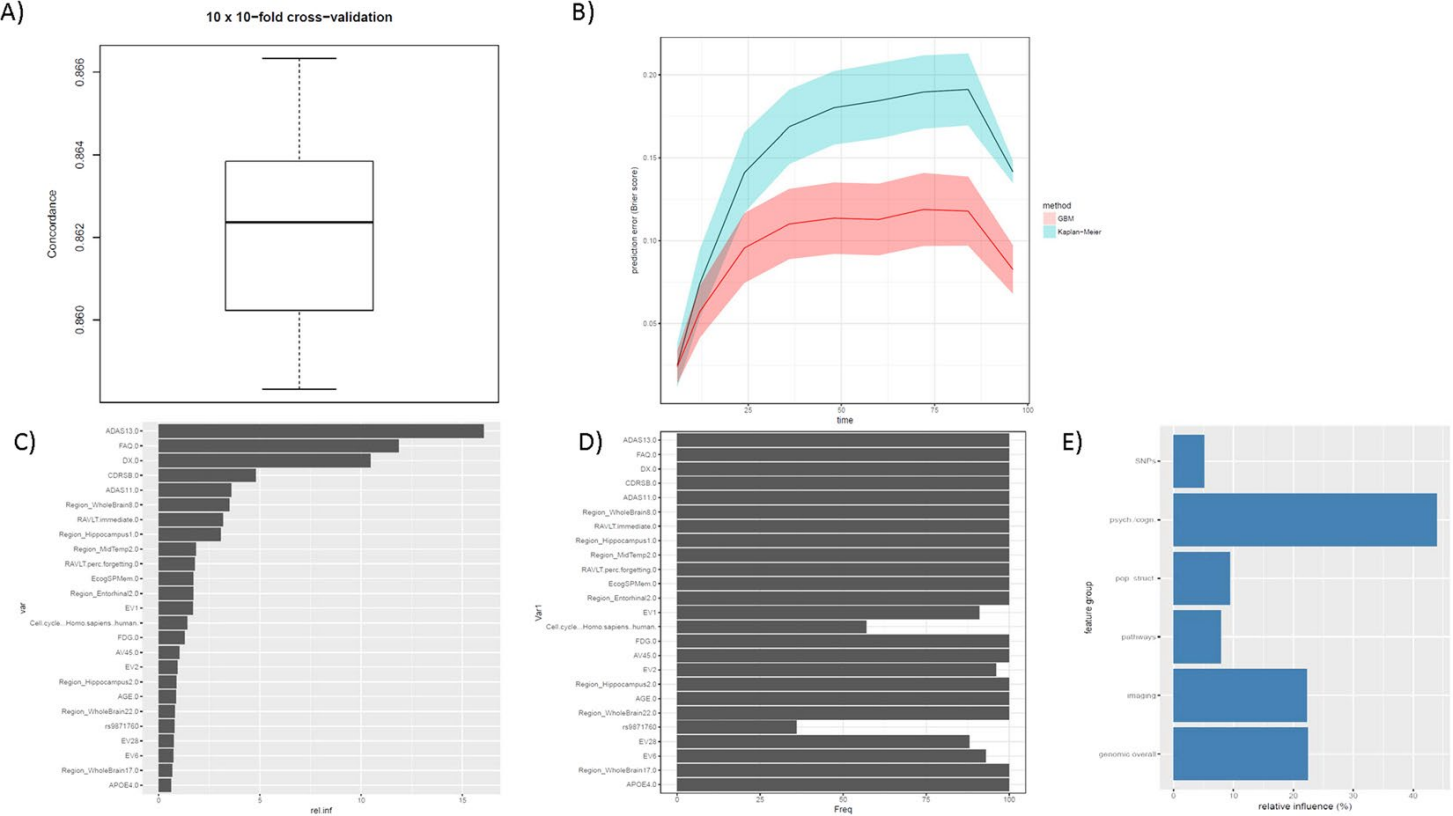
(**A**) Boxplot of cross-validated concordance index. (**B**) Prediction error (Brier score) as a function of time for GBM vs. Kaplan-Meier estimator. The prediction error curve is calculated on held out test data during the 10 times repeated 10-fold cross-validation procedure. The solid curve corresponds to the mean and the shaded area to the standard deviation. (**C**) 25 most relevant features according to GBM model trained on the whole tuning dataset. (**D**) Selection frequency of these features during the 10 times repeated 10-fold cross-validation procedure. (**E**) cumulative relative influence of feature groups in final model.

As indicated in Fig. 2A our algorithm achieved a high prediction performance with a cross-validated C-index of 0.86. Figure 2B depicts the time dependent prediction error (in terms of Brier score) of the GBM model on held out test data during the repeated cross-validation procedure in comparison to a Kaplan-Meier estimator, showing clearly superior performance with low prediction error. We thus conclude that our employed multi-scale data allows for an accurate prediction of the time dependent risk to convert from normal/MCI to AD pathology. Notably, our developed GBM model achieved a significantly higher cross-validated C-index than another and popular ensemble based decision tree technique, Random Survival Forest^15^, elastic net penalized Cox regression^16,17^ and two different Canonical Correlation Analysis (CCA) based methods^18,19^ followed by conventional Cox regression, see Methods.

#### Most Relevant Features are Interpretable

To better understand the contribution of individual features for the AD risk prediction we ranked variables according to their relative importance in a final GBM model that was trained on all available data (Fig. 2C). The top 25 most relevant variables comprised, besides baseline diagnosis (DX), results of different neuro-psychological/cognitive assessments (Alzheimer’s Disease Assessment Scale Cognitive Plus - ADAS13, ADAS11, Functional Assessment Questionnaire - FAQ, Clinical Dementia Rating - CDRSB, Rey Auditory Verbal Learning Test - RAVLT, Everyday Cognition Study Partner Report - EcogSPMem), neuro-imaging features (Region_Hippocampus, Region_Enthorhinal, Region_MidTemp, Region_WholeBrain8), PET and FDG PET imaging diagnosis (AV45, FDG), APOE4 status as well as patient age. Furthermore, different features describing the genetic population sub-structure (EV1, EV2,…) as well as the SNP functional impact on cell cycle were contained. It has been suggested that dysfunction in neuronal cell cycle reentry plays a fundamental role in AD pathology^20^. More specifically, the hypothesis has been stated that the disease is caused by aberrant re-entry of different neuronal populations into the cell division cycle^21^.

Notably, most of the top 25 were selected highly stable during the 10 times repeated 10-fold cross-validation procedure (Fig. 2D). That means the vast majority of GBM models trained during the cross-validation procedure contained the same most relevant features. This finding specifically includes the above mentioned cell cycle. Altogether there were 170 featues that were selected at least in 50 out of 100 times (see full list in Supplementary material). These features contained the neuro-psychological assessments (ADAS, Ecog, RAVLT, CDRSB, MMSE, FAQ), PET scanning results (AV45, FDG), APOE4 status, age, baseline diagnosis, educational status as well as different brain regions and pathways (including cell cycle). The most stably selected SNP rs10509663 (selected 70/100 times) has been associated with CSF levels of amyloid-*β*. Misfolding of this peptide is a well known hallmark of AD that results into the characteristic plaques in the brain of AD patients^22^. Interestingly, immune system and ribosome were found as most stably selected pathways (84/100 times). It has recently been indicated that activation of the innate immune system plays a crucial role in disease progression^23^. Ribosome dysfunction has been observed as an early event in AD development^24^.

The most influential SNP in the final GBM model was rs9871760 (selected 36/100 times), which has been associated to the whole brain volume^25^. The TT or CT genotypes of the second most relevant SNP rs3756577 (CAMK2A, selected 32/100) have been associated with a nearly 8 times risk reduction for AD^26^. Two other examples include rs4263408 (selected 32/100 times) and rs6859 (selected 60/100 times). The SNP rs4263408 (UBE2K) has been found to affect amyloid-*β* concentrations^27^. The SNP rs6859 (NECTIN2) has been associated with late AD onset^28^.

Altogether the cumulative relative influence of all genomically derived features (including APOE4 status) was ~22% in our model, and 109/170 features that were selected at least 50/100 times during the repeated cross-validation procedure were genomically derived. Figure 2E systematically visualizes the cumulative relative influence of different feature groups, such as SNPs, neuro-psychological/cognitive tests, features describing the genomic population sub-structure (principal components), SNP impact on pathways and neuro-imaging features. This demonstrates an equal contribution of neuro-imaging and genomic features, whereas neuro-psychological/cognitive test results have an almost twice has high cumulative influence.

#### The Model Allows for Patient Stratification

**Figure 3:**
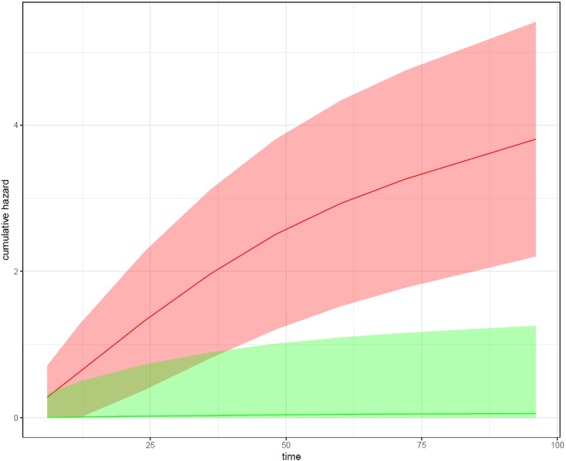
Cumulative hazard as a function of time for the 10% patients with highest AD risk scores (red) and 10% patients with lowest AD risk scores (green). Depicted are the average risk curves plus standard errors as confidence bands.

Figure 3 exemplifies the possibility to stratify patients by the predictions made by our model into “high risk” and “low risk” groups. More specifically the Figure compares the cumulative risk curves of 93 patients in the upper 10% quantile of the risk score produced by our model with 93 patients in the lower 10% quantile of the risk score. Both curves show a clear difference (*p* ≈ 0, log rank test). We performed univariate statistical tests (Wilcoxon for continuous and *χ*^2^-test for discrete variables) for each individual feature used in our GBM model to better understand differences between the high risk and low risk group. P-values were corrected for multiple testing using the Benjamin-Yekutieli false discovery rate (FDR) control under dependency^29^. Accordingly, we found clear differences in the APOE4 status (*FDR* < 1*e*−9), in all neuro-psychological assessment scores, PET imaging diagnosis (*FDR* < 1*e*−4), rs405509 (*FDR* < 0.05), ErbB signaling and olfactory transduction (both *FDR* < 0.05). In the low risk group 63% of the patients were diagnosed as healthy at baseline, whereas in the high risk group all patients were already late phase mild cognitively impaired. According to dbSNP^30^, rs405509 is located in the APOE4 gene region and synergizes with the APOE4 *ε*4 allele in the impairment of cognition. The T allele has been identified as a risk factor for AD^31^. ErbB signaling and olfactory transduction both showed a significant difference in SNP pathway scores. However, the difference in the impact score was in both cases less than 1%. Hence, any further interpretation should be taken with care. However, we like to mention that olfactory dysfunction and insufficient ErbB signaling have both been associated with AD^32,33^.

### Bayesian Network Modeling Reveals Dependencies Between Relevant Features

Our predictive GBM model altogether contained a set of 335 features. To gain a better understanding of the complex and multiple interactions between these features we developed a Bayesian Network (BN) model^34^. BNs belong to the family of probabilistic graphical models and enable the description of a complex multivariate distribution with many variables (here all relevant features in our predictive model). BNs can be visualized as graphs, where nodes correspond to random variables and edges reflect conditional statistical dependencies. BNs have a long tradition in systems biology for learning and describing biological pathways^35–37^, because they - at least partially - allow for discovery of causal relationships from observed data (see Methods).

**Figure 4:**
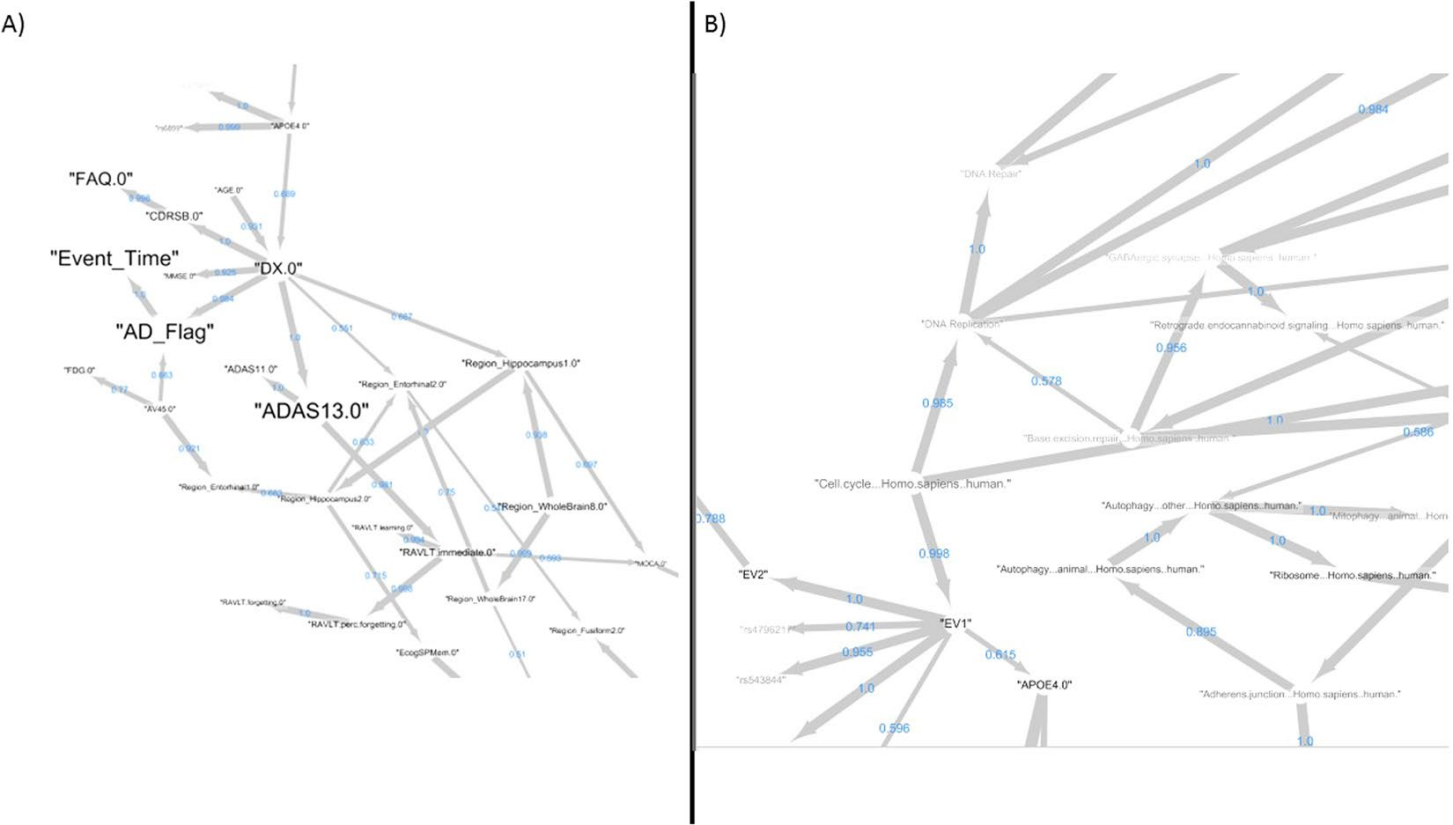
Edges appearing in more than 50% of 1000 Bayesian Network reconstructions based on random sub-samples of the data. Line thickness is proportional to the relative frequency of observing an edge in the 1000 network reconstrucions, and the corresponding number is shown as edge label. The node size is proportional to the relative influence of the variable in the final GBM model, and the color reflects the selection frequency in the repeated cross-validation procedure (more black = higher stability). Sub-figures (**A**) and (**B**) depict two examples zooms into the overall network.

We developed a BN for the same 924 patients used in our final predictive GBM model. Importantly, we included the time until AD diagnosis together with a censoring indicator as variables. Six different BN learning algorithms were compared via a 10-fold cross-validation procedure (see Methods) and the best performing one (tabu search^38^) selected. Subsequently, we applied a non-parametric bootstrap to network learning, resulting into 257 edges appearing in more than 50% of 1000 network reconstructions based on random sub-samples of the data (see details in Methods section). Figure 4 shows two zooms into the network of these stable edges highlighting the direct dependency of the clinical outcome (Event_Time, AD_Flag) on baseline diagnosis (DX) and PET scan (AV45). PET scanning results manifest in the entorhinal region, which is known to be affected by AD pathology^39^. Baseline diagnosis is dependent on age and APOE4 mutation status. APOE polymorphic alleles are one of the major AD risk factors^40^. Baseline diagnosis influences neuro-psychological assessments (ADAS13, ADAS11, MMSE) and manifests in the entorhinal region and hippocampus, which is vulnerable specifically in early AD stages^41^.

Figure 4B further highlights the dependency of APOE4 status on sub-population structure (EV1), which is reflected by differences in cell cycle, hence supporting the above cited neuronal cell cycle hypothesis as one of the possible disease causes. Cell cycle includes DNA replication and repair, which is mirrored by a corresponding edge in our BN. DNA damaging by oxidative stress has been reported as one of the earliest detectable events in the progression to dementia^42^. More specifically, altered DNA repair has been observed in GABAergic neurons^43^ and let to the idea of a therapeutic modulation of the GABAergic system in early AD stages^44^. GABAergic neurons distinctly express *CB*_1_ receptors, thus explaining the subsequent link to the endocannabinoid system^45^. Targeting this system has been discussed as a therapeutic option^45^.

To specifically validate some of the less obvious dependencies between pathways that were reflected via stable edges in our BN we checked the overlap of genes that could be mapped to the respective pathways based on KEGG^46^ and Reactome^47^ databases. The statistical significance of overlaps was assessed via a hyper-geometric test, and p-values corrected for multiple testing via the Benjamini-Yekutieli false discovery rate (FDR) under dependency^29^. Accordingly we obtained significant results (*FDR* < 5%) for 63/76 (83%) pathway pairs in our BN (see results in Supplements).

### Pathway Dependencies are Interpretable via Biological Mechanisms

#### Mapping of Stable Edges to Causal Biological Mechanisms

To further validate pathway dependencies found by our BN methodology and to gain additional insights into underlying molecular mechanisms we employed a literature derived mechanistic AD disease model encoded in the OpenBEL language^48^. Briefly, this model describes cause-effect relationships between different biological entities, such as genes, SNPs and biological processes (e.g. neuronal death) in a purely qualitative manner. We developed an algorithmic approach to map nodes and edges in our network to the OpenBEL AD disease model. In conclusion, we could identify 12 cause-effect-relationship networks that could be linked to specific stable pathway-pathway edges in our BN (see Figures in Supplements). We have developed a software tool to explore our BN and associated mechanism mappings in a fully interactive manner. The tool is accessible under http://neurommsig.scai.fraunhofer.de/bayesian. In the following we discuss two selected mechanism mappings in greater detail.

### Example 1: Adherens Junction and Autophagy

**Figure 5:**
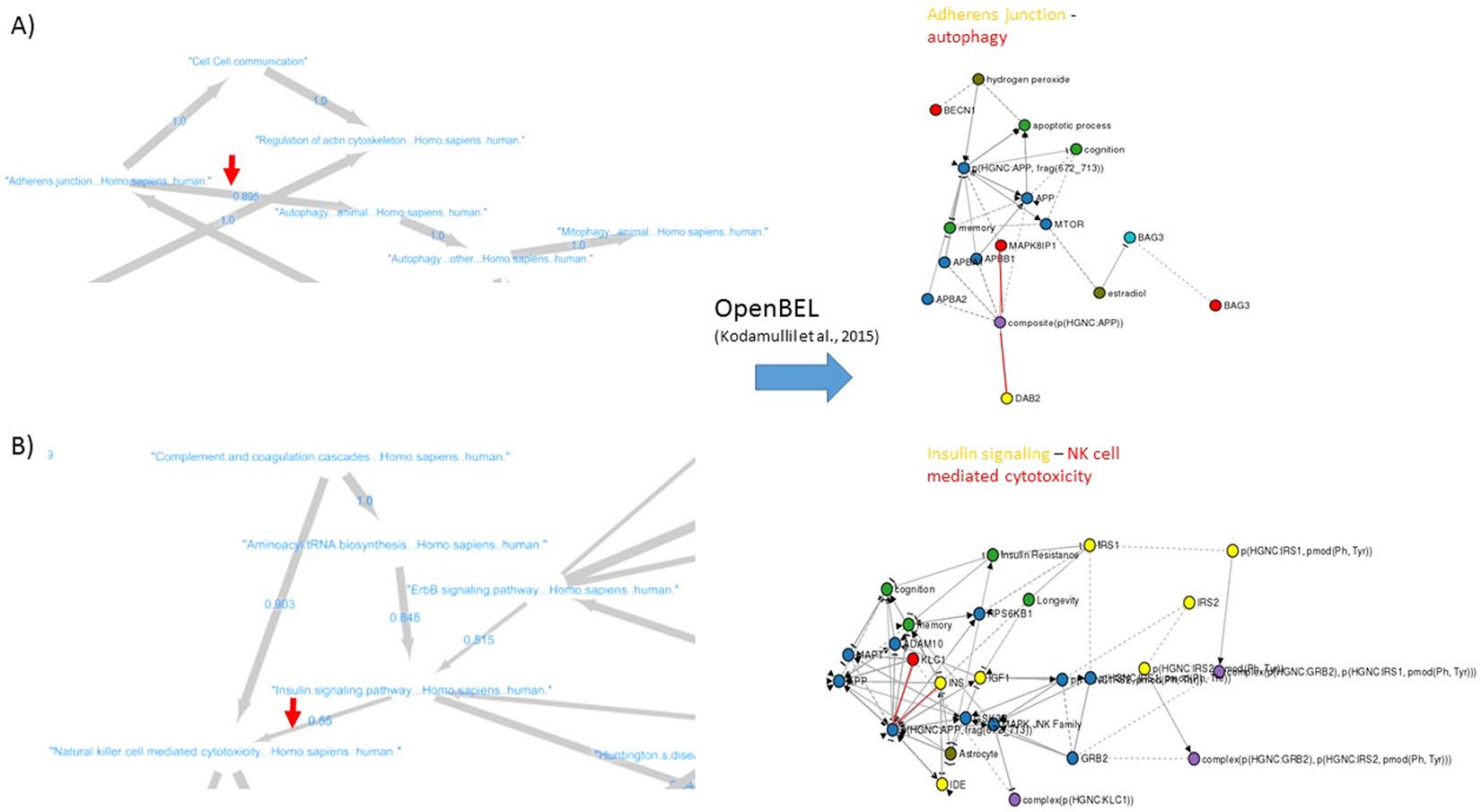
Two examples of mapping stable BN edges to biological mechanisms via the OpenBEL AD graph by Kodamullil *et al*.^48^: (**A**) adjherens junction and autophagy; (**B**) insulin signaling and natural killer cell mediated cytotoxicity. Biological entities mapping to the source of the edge marked by an arrow on the left hand side of the Figure are drawn in yellow in the OpenBEL graph on the right hand side. Biological entities mapping to the sink of the edge are shown in red. Red edges highlight the shortest among all possible paths connecting yellow and red nodes.

As a first example, our Bayesian Network predicts an association between adherens junction and autophagy, which has recently also been proposed in Nighot *et al*.^49^ (Fig. 5A). Adherens junctions, also known as tight junctions, are comprised of epithelial cells that are present in all tissues, particularly in junction between cerebral epithelial cells of the blood-brain barrier (BBB)^50,51^. The BBB is a biochemical barrier which regulates the entry of blood based molecules into the brain and helps in maintaining ionic homeostasis within the brain. These barriers further inhibit diffusion of cellular components thereby protecting the central nervous system^52,53^. However, during pathological conditions such as AD, the BBB are disrupted increasing the cell permeability as well as accumulation of amyloid-*β* resulting in autophagy.

Mapping of the edge between adherens junction and autophagy in the BN to OpenBEL encoded mechanisms allowed us to identify molecular players, which may play a role in the normal/MCI to AD transition: Proteolytic processing of amyloid precursor protein (APP) is one of the hallmarks of AD pathophysiology^22^. The processing of APP to amyloid-*β* is greatly affected by the sub-cellular localization of *β* and *γ* secretases due to trans-membrane receptors as well as adapter proteins^54,55^. Internalization of APP via adapter proteins such as DAB2 triggers clathrin mediated endocytosis by binding to the YXNPXY motif region of APP triggering endocytosis^56,57^. Apart from DAB2, there are other adapter proteins that trigger the production of amyloid-*β* namely APBA2 and APBA1. These two proteins are enriched in neurons and contain a phosphotyrosine binding site (PTB) domain^58,59^. These proteins are involved in cellular activities pertaining to neuronal transport and synaptic function. Unlike DAB2, APBA1 and APBA2 interact with the YENPTY motif region of APP and thereby affecting APP trafficking. During AD pathology, APBA1 protein modulates the secretory and endocytic trafficking of APP whereas APBA2 accelerates APP endocytosis which leads to autophagosomes that enhances amyloid-*β* internalization^60,61^.

Autophagosomes are structures that facilitate the break down of accumulated amyloid-*β* peptides by fusion with lysosomes. Lyosomes contain enzymes that break down accumulated peptides^62,63^. However, during AD progression, autophagosomes accumulate within neurons of AD patients. The scaffolding protein MAPK8IP1 is a regulator of autophagosomal motility by activating the c-Jun-N-terminal kinase (JNK), which mediates the JNK signaling cascade. JNK signaling formulates the formation of neurofibrillary tangles through direct phosphorylation of tau proteins further resulting in stress induced apoptosis in neuronal cells^64,65^. Furthermore, the activation of JNK signaling induces phosphorylation of Bcl-2 releasing beclin-1 protein which further aggravates autophagosome formation, resulting in cognitive decline^66,67^.

### Example 2: Insulin Signaling and Natural Killer (NK) Cell Mediated Cytotoxicity

Another example is the BN predicted link between insulin signaling and natural killer (NK) cell mediated cytotoxicity (Fig. 5B), which has again been proposed in the literature^68^. Our extracted mechanism shows, how the axonal transport and APP trafficking may influence AD development: APP proteins are transported to distinct nerve cells through axons via anterograde pathways for maintaining homeostasis and neuronal function^69^. The fast anterograde transport is mediated through APP and Kinesin 1 (KLC1) and Fe65, adapter protein. The proteolytic processing of amyloid-*β* occurs within the axons and through this process amyloid-*β* is generated releasing the complex KLC1 from APP. During AD progression, the excessive production of amyloid-*β* prevents the release of Kinesin and thereby restricts the axonal transport. The arrested axonal transport also triggers the phosphorylation of APP through the amyloidogenic pathway concomitantly releases the Fe65 and translocates into the nucleus to regulate the expression of stress-related genes including glycogen synthase kinase 3 beta (GSK3B)^70,71^.

Apart from APP and KLC1, insulin and IGF regulate neuronal stem cell activation, synaptic maintenance and neuroprotection^72,73^. Insulin regulates the glucose and lipid metabolism in the brain and thereby contributes to learning and memory^74^. It is known that insulin is locally produced in the brain and can be easily transported through the BBB^75–77^. The glucose metabolism is mediated by binding of the IGF to its receptor promoting the phosphorylation of the tyrosine residue and further phosphorylating the insulin receptor substrate (IRS) at the tyrosine residue. The two receptors, IRS-1 and IRS-2 are mediators of insulin-dependent mitogenesis and regulation of glucose metabolism, which is a part of the insulin-signaling pathway^78,79^. The phosphorylation of IRS1 results in the downstream activation of AKT, mammalian target of rapamycin (mTOR), growth receptor binding protein 2 (GRB2), mitogen-activated protein kinase (MAPK) and GSK3B, thereby promoting the APP transport and clearance of amyloid-*β* from the BBB^80,81^. During AD progression, the insulin signaling pathway shows aberrant activity, resulting in increased accumulation of amyloid-*β*, tau phosphorylation and decreased cerebral blood flow. Furthermore, the binding of IRS to its receptor is inhibited, resulting in decreased glucose metabolism and cognition^82^. Brain insulin resistance thus contributes to AD, a complex phenomenon accompanied by IGF-1 resistance and dysfunction of IRS-1 triggered by amyloid-*β* oligomers, stimulating cognitive decline independent of AD pathology^83^.

## Discussion

Determining the risk of an individual to develop AD is an important aspect to start treatment with disease modifying drugs as early as possible and to better manage the disease. To better address this need we developed a highly predictive time-to-event model for normal/MCI to AD transition based on multi-modal data from ADNI. For this purpose we proposed a novel approach to capture the functional impact of SNPs on pathway level for each individual patient. Analysis of our model confirmed the significant impact of the baseline diagnosis (cognitively normal, early or late stage mild impaired) for predictions and demonstrated the crucial role for stratifying patients into high and low risk groups. Further relevant features of our model include neuro-psychological assessment scores, neuro-imaging features (including PET scan results), age as well as genetic predisposition, which altogether contributed 22% cumulative influence. In addition to well known risk factors such as APOE4 status we specifically identified SNP functional impact on cell cycle as a relevant feature in our model, which agrees well with the hypothesis of AD being caused by dysfunction of the neuronal cell cycle reentry^20^.

As a further contribution of our work we tried to better understand dependencies of relevant features via a Bayesian Network model. Thanks to our proposed pathway functional impact score and with the help of a specifically developed algorithm we were able to relate several non-obvious links to detailed biological mechanisms, which constitutes a partial literature based validation. As two examples we discussed the mechanisms linking tight junction and autophagy as well as insulin signaling and NK cell mediated cytotoxicity in more detail and provided literature based evidence for the involvement into AD pathology. Altogether stable edges found in our BN provide a broad overview about the complex interplay of different AD risk factors and provide insights into their underlying biological mechanisms. Such insights could potentially help in developing novel and more mechanistic therapies in the future, which are critically needed in the field.

Of course, there are limitations of our work that we would like to mention: The whole work presented here was based on the ADNI cohort. Since this patient group primarily represents an amnestic rather than an epidemiologically selected population we cannot exclude population biases. Hence, a confirmation of our findings based on a different study cohort has to be conducted in the future. Furthermore, it is believed that AD pathology starts decades before actual diagnosis^84^. The age range of ADNI subjects is thus probably too late to find very early disease indications, and the follow-up time of 96 months is likely too short for many patients that were initially cognitively normal to allow for a definite AD diagnosis till end of study. Taking these aspects into consideration we therefore see the need for long lasting studies in a more epidemic population in the future. Key findings from our work could help designing such a study by identifying relevant factors to measure.

## Methods

### Clinical and Genomic Feature Extraction from ADNI

#### Data Preprocessing

Clinical variables (including age, gender, education level, neuro-psychological assessments, pre-computed volume measurements of different brain regions and PET scan results) from all ADNI studies (ADNI1, ADNI2, ADNI-GO) were retrieved via the ADNImerge R-package (https://adni.bitbucket.io/), altogether comprising 73 features at study baseline after dropping variables with more than 65% missing values. For the 818 subjects in the ADNI1 study population 620,901 SNP calls were available via the Illumina Human610-Quad BeadChip platform. Further genomic data (730,525 SNPs, Illumina HumanOmniExpress BeadChip) was available for 432 subjects in ADNI2/GO. 979 patients were diagnosed as either normal or MCI at baseline, and 314,134 SNPs were found in common between both ADNI phases. Following common convention we encoded SNPs by the occurrence of a minor allele (0, 1, 2) while taking dbSNP as ref.^85^.

The initial set of 979 patients was reduced to 926 by filtering out individuals with kinship coefficient <0.1 and inbreeding coefficient >0.1. Kinship coefficient was calculated by the PLINK method of moment for identity-by-descent analysis^86^. Inbreeding coefficient estimation was based on the method described in Yang *et al*.^87^. For both analyses we employed the SNPRelate software^88^. SNPs with MAF <1% or missing rate >5% were filtered out.

#### Literature Derived SNPs

**Figure 6:**
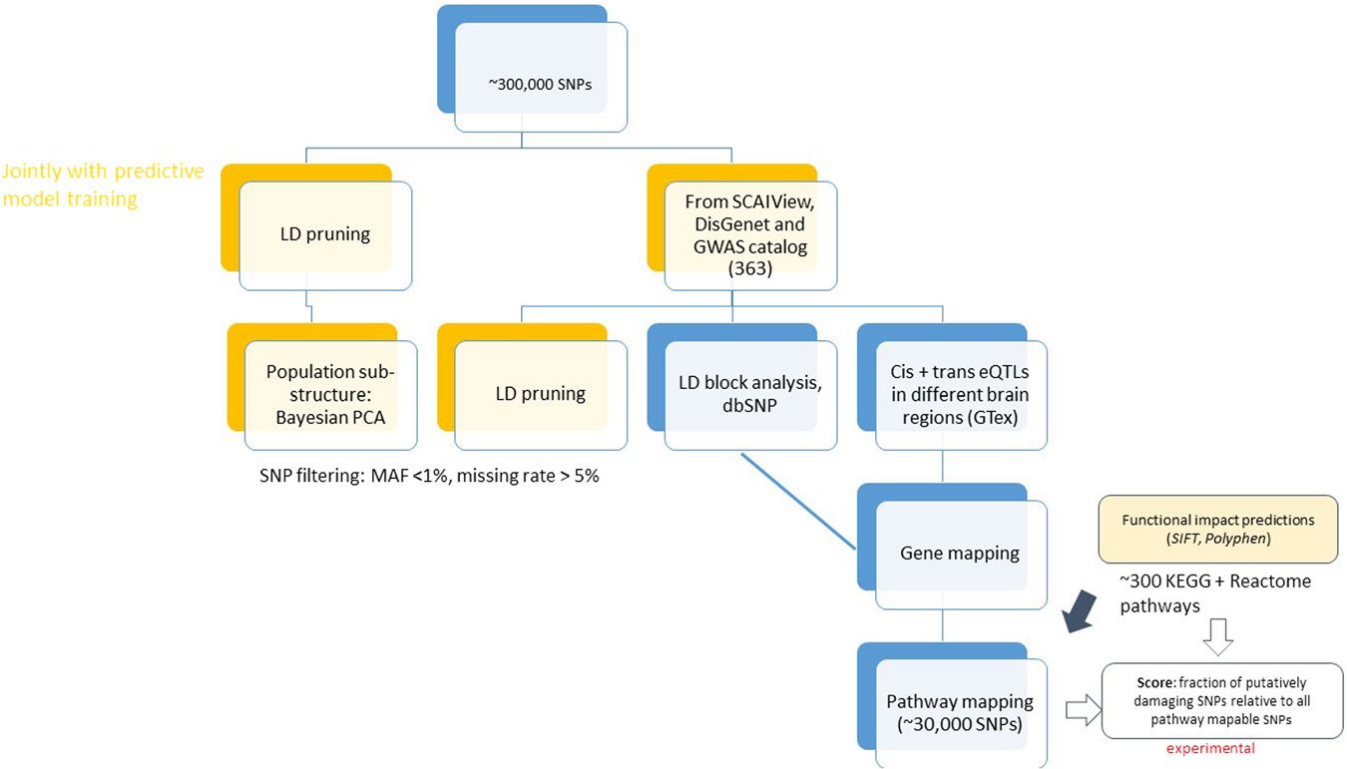
Approach to genomic feature extraction.

We used the text mining software SCAIView^89^, the DisGeNET database^90^ and GWAS catalogue^91^ with a similar search query (“Alzheimer’ s Disease” and “Homo sapiens”) to retrieve 1,866 putatively disease associated SNPs, of which 363 intersected with the 314,134 SNPs measured in both ADNI phases (Fig. 6). Application of LD pruning (*r*^2^ < 0.2) using SNPRelate reduced this number further to ∼300. Notably, this step was done as part of predictive model training and more specifically also within the repeated cross-validation procedure.

#### Principal Components

In addition to knowledge derived, LD pruned SNPs, we considered the global genetic population structure by retrieving the top principal components (based on all 314,134 SNPs) via a Bayesian principal component analysis (PCA)^92^ after LD pruning. We favored Bayesian PCA over conventional maximum likelihood based PCA here due to the high dimensionality of SNPs compared to the number of patients. Bayesian PCA effectively regularizes PCA and thus results into more stable and robust estimates. Again, Bayesian PCA was done as part of model training and thus within the repeated cross-validation procedure. Note that the extremely high dimensionality of SNP data prevents us from extracting all principal components due to prohibitive computational costs. However, extraction of the top *k* (here: 32, the default in SNPRelate) principal components can be done efficiently using Lanczo’s method^93^. We relied on the implementation provided in the SNPRelate software^88^. The proportion of explained variance by the top principal components was typically around 5–6%, depending on the actual set of patients in the training set. This fact highlights that the top principal components for sure do not describe the global population structure entirely. However, they may still capture useful signals for our predictive model. The fact that several eigenvectors were among the most relevant features (Fig. 2) in our final model supports this thought.

#### SNP Based Pathway Impact Scores

We performed a gene mapping of SNPs. This was done by

1. Considering all 363 putatively disease associated SNPs taken from the literature and all those in strong LD (*r*^2^ > 0.8). Each SNP in an LD block was then mapped to its closest genes via dbSNP^30^.
2. Considering significant (false discovery rate <5%) cis- and trans-eQTLs in different brain regions from GTex^94^. For this step the same SNPs as in the previous one where used.

Note that both steps can result into a mapping of one SNP to several genes. The The entire set of genes was then extended to a pathway mapping, where pathways were taken from KEGG^46^ and Reactome^47^. Around 30,000 SNPs were map-able to pathways altogether. For these ∼30,000 SNPs we used functional impact predictions from SIFT^95^ and Polyphen2^96^. We then developed an experimental score to capture the overall functional impact on each pathway per patient: This score was defined as the fraction of at least possibly damaging/deleterious SNPs relative to all pathway map-able SNPs. This score is a number between 0 and 1 for each individual patient and pathway.

### Multi-Modal Time-to-AD-Diagnosis Prediction

#### Intermediate Data Fusion with Gradient Boosting Machines

The employed data is characterized by a high heterogeneity with respect to different statistical distributions and numerical scales (e.g. SNPs vs. neuro-imaging features). Gradient Boosting Machines (GBM) have been introduced as a decision tree based ensemble learning technique that enables non-linear and non-parametric time-to-event prediction based on such heterogeneous data^12^. A GBM constitutes a weighted ensemble of weak decision tree classifiers (base learners) with restricted maximal depth (here 3). A higher maximal tree depth results into more complex base learners. that capture higher order interactions between variables (here: up to 3-way interactions). On the other hand, a tree depth of 1 corresponds to simple decision stumps and can require longer boosting, depending on the overall optimal complexity of the GBM model. The reason is that the actual number of trees in the ensemble (and thus overall complexity of the GBM model) critically depends on the number of boosting steps, which is a tunable hyper-parameter. Depending on the maximal depth and number of decision trees GBMs do not necessarily employ all existing features in the data, but possibly only a subset. We found the optimal number of boosting steps found via an inner 10-fold cross-validation. Importantly, this was done within the outer 10 times repeated 10-fold cross-validation procedure used to evaluate prediction performance.

GBM can deal with censored time-to-event (here: time to AD diagnosis) data, as in our application: We like to predict the time until AD is diagnosed. For some AD converters such a diagnosis will be observed within the study time. However, there are also patients for which the diagnosis cannot be established within the study time, but potentially after the end of study. Their observed times to event are thus right censored. Our employed GBM implementation (R-package gbm) allows for dealing with time-to-event data by using the negative partial log-likelihood of the Cox proportional hazards model as a loss function.

As typical in clinical studies ADNI data contains missing values. GBM rely on a surrogate split approach for this purpose^97^. GBM allow for a ranking of variables according to their relevance for the model. This is done by recording the relative reduction of the error loss function as a measure of variable importance. Accordingly, features with zero importance can be filtered out. Hence, GBM can be used for feature selection.

In our data there is a difference in the number of features from SNPs, pathways, principal components and clinical data. In order to avoid any potential selection bias towards one of these feature types due to differing number of features we decided to implement a two-step strategy:

1. Training of a separate GBM model for each data modality and selection of most relevant features.
2. Joining of these features and training of a final GBM model.

Note that the first step ignores feature dependencies from different modalities while the second one takes them into account.

Multi-modal data fusion is a field of active research, and there is not a universal best performing approach. In the data science literature classically three general strategies for multi-modal data fusion are distinguished^98,99^, see Ahmad and Fröhlich^100^ for a more extensive review^100^. Early data fusion methods focus on extraction of common features from several data modalities, resulting into one integrated data matrix. In a second step conventional machine learning methods can then be applied. Late integration algorithms first learn separate models for each data modality and then only combine predictions made by these models, for example with the help of a meta-model trained on the outputs of data source specific sub-models. These methods hence ignore feature dependencies between different data modalities. Intermediate integration algorithms are the youngest branch of data fusion approaches. The idea is to join data sources while building the predictive model. Our proposed approach can be seen as an instance of an intermediate data fusion approach.

No significant difference in prediction performance (C-index) compared to a conventional approach using a GBM model with all features was observed (*p* = 0.35, Wilcoxon test). However, the final GBM model with the two-step strategy contained fewer features (335 vs. 435).

#### Comparison to Other Prediction Methods

We compared our proposed approach to a Random Survival Forest^15^, resulting into a significantly higher C-index with our GBM modeling strategy (*p* = 1.1*e*−5, Wilcoxon test, Figure S4). The GBM model also outperformed an elastic net penalized Cox regression^16,17^ (*p* = 0.0002, Wilcoxon test, Figure S4). Importantly, application of elastic net penalized Cox regression requires to impute missing values. This was done via the missForest algorithm^101^ in a pre-processing step prior to running the cross-validation. Note that this step may result into slightly over-optimistic results for the elastic net.

In addition, we compared our approach against supervised sparse Generalized Canonical Correlation Analysis (ssGCCA)^18,19^ in conjunction with a conventional Cox regression as predictive model. Sparse GCCA is an early data fusion approach that extracts latent variables (canonical variates) from different data modalities (here: clinical, SNPs, pathways, principal components). Each canonical variate describes a sparse linear combination of existing features within a specific data modality and is chosen to maximize the sum of correlations with canonical variates from other data modalities. We used a sparse GCCA version here, because we expect only a subset of original features to be relevant for the predicted outcome. In addition, we performed a supervised pre-filtering of features in each data modality. For this purpose and in agreement to Witten *et al*. we performed univariate Cox regressions and selected the 20% features with lowest p-value according to the log-likelihood ratio test. Afterwards we conducted feature extraction via sparse GCCA, as described before, and projected data of each modality into the low-dimensional space spanned by first canonical variates, and in that space a predictive Cox regression was trained. We here tested two different sparse GCCA implementations, one provided in R-package “mixOmics”^102^ and the other one provided in R-package “PMA”^18^. Sparse GCCA involves tuning of regularization/sparsity parameters for each data modality. The sparse GCCA implementation in R-package “PMA” provides a permutation test for this purpose, while the sparse GCCA implementation in R-package “mixOmics” requires to run an inner cross-validation over a grid of regularization parameters (see details in Supplements). Both ssGCCA methods performed similar and resulted into significantly lower C-indices than our GBM approach (median 80% with PMA, 82% with mixOmics vs. 86% with our method; *p* < 1*e*−4 for PMA and mixOmics vs. our method with Wilcoxon test, see Figure S4). Omitting the supervised pre-filtering step suggested by Witten *et al*. resulted into a clear drop of the C-index by around 5% (*p* = 1.8*e*−5 with Wilcoxon test, see Figure S4).

### Bayesian Network Learning

#### General

Let *G* = (*V*, *E*) be a directed acyclic graph (DAG) and *X* = (*X*_*v*_)_*v*∈*V*_ a set of random variables indexed by nodes in *V*. *X* is called a Bayesian Network (BN) with respect to *G*, if the joint distribution *p*(*X*_1_, *X*_2_, ..., *X*_*n*_) factorizes according to:

1 $$p({X}_{1}={x}_{1},{X}_{2}={x}_{2},\ldots ,{X}_{n}={x}_{n})=\prod _{v\in V}p({X}_{v}={x}_{v}|{X}_{pa(v)}={x}_{pa(v)})$$

where *pa*(*v*) denotes the parents of node *v* and ***x***_*pa*(*v*)_ their joint configuration^34^.

The Markov Blanket (MB) of node *v*, *MB*(*v*), is defined as the set of nodes consisting of *v*’s parents, children, and any other parents of *v*’s children. If *X* is a BN with respect to *G*, then every node is conditionally independent of all other nodes in the network, given its Markov Blanket, i.e.

2 $${X}_{i}\perp {X}_{j}|{X}_{MB(i)}\,{\rm{for}}\,{\rm{all}}\,i\in V,j\in V-\{i\}-MB(i)$$

#### Learning the Structure of a Bayesian Network from Data

In the simplest case the DAG *G* is defined by an expert, but in many real life applications (as our present one) this is not the case and thus *G* should be learned from data. In general there exists two existing strategies for that purpose: search-and-score and constraint-based algorithms^34^. Search-and-score based approaches walk through the space of all possible DAG structures and score each candidate by its fit to the data. Typically such methods are thus computationally not scale-able to large BNs. In contrast, constrained-based approaches are significantly faster and scale-able to BNs with hundreds of variables. They typically rely on conditional independence tests between variables^103^. In this work we used and compared six different algorithms implemented in the R-package *bnlearn*^104^: greedy hill climbing (50 random restarts), tabu search^38^, Max-Min Hill Climbing (MMHC)^105^, Max-Min Parent Child (MMPC)^105^ and semi-interleaved Hiton Parent Child (SI-HITON-PC)^106^. Greedy hill climbing and tabu search are heuristic score based optimization approaches, whereas MMPC and SI-HITON-PC are constrained-based structure learning methods that try to identify the Markov Blanket of each node in the Bayesian Network. MMHC is a hybrid approach, which uses ideas from both, search-and-score as well as constrained-based techniques: MMHC first learns the skeleton of the BN using the MMPC constrained-based algorithm. In a second phase edges are then oriented via a greedy hill climbing search.

Selection between different BN structure learning algorithms can be done via *k*-fold cross-validation akin to conventional supervised learning^34^. That means the overall data is randomly split into *k* folds, and the BN structure together with its parameters successively learned from *k*−1 folds. If the fitted BN correctly models the overall population (and not just the training data), the data in the left out fold should with high probability fall into the same statistical distribution that is described by the BN. This can be quantified via the negated expected log-likelihood of the test data. Accordingly, cross-validation can be used to assess the generalization ability of a BN model and to compare different structure learning algorithms on that basis (see results in Supplements).

An important question in BN structure learning is, in how far the learned structure reflects causal relationships in the data. Indeed, if the BN is faithful to the underlying statistical distribution (i.e. models it correctly), then the true causal network is known to be part of a class of equivalent graph structures, called *class partially directed acyclic graph* (CPDAG)^34,103^. Under the above mentioned assumptions the CPDAG has the same skeleton as the true causal graph, but may leave some edges undirected. Hence, in practical applications it is important to restrict the CPDAG equivalence class as much as possible by prior knowledge to allow correct orientation of as many edges as possible. In our case we specifically imposed the following constraints for BN structures:

- No genomic feature can be influenced by a non-genomic feature. However, genomic features are allowed to have interactions among themselves (e.g. pathway-pathway dependencies).
- Neuro-psychological test results cannot be influenced by neuro-imaging features, but the other way around is possible.
- Age does not depend on any other variable.
- The baseline diagnosis cannot be influenced by any clinical variable, except for age and education level.
- The education level does not depend on any other clinical variable.
- The time-to-AD diagnosis is always dependent on a censoring indicator, and it does not influence any other variable.

Despite of these constraints identifying the true (CP)DAG structure from limited data still remains a challenge and thus raises the question, how confident one can be about the existence of an individual edge. One possible way of addressing this question is via a non-parametric bootstrap^107^. Briefly, given data from *N* patients we sample *N* patient records with replacement for a number of times (here: 1000). For each of these 1000 bootstrap samples a BN structure is learned. Afterwards the relative frequency of observing a particular edge is recorded, resulting into a confidence measure, which reflects the robustness of an edge against perturbations of the data.

Prior to BN structure learning we imputed missing values on the whole dataset of 900 patients using the missForest algorithm for mixed categorical and continuous data types^101^. Furthermore, all variables were discretized into three bins using equal interval width.

### Comparison Against Literature Derived Cause-Effect-Relationships

We refer to^48^ for details about the construction of the literature derived, cause-and-effect relationship model for AD. One of the main challenges with this model is that it contains many variables that have no direct correspondence in our data and vice versa. In our case only SNP rs405509 (in APOE4 gene) could be mapped directly to the OpenBEL model. To address this challenge we employed NeuroMMSig^108^. NeuroMMSig categorizes biological entities according to their role in disease specific pathways based on support from the literature. Corresponding references are stored in the NeuroMMSig database. This gene set view allowed us to relate OpenBEL sub-graphs to KEGG and Reactome pathways. For this purpose each KEGG and Reactome pathway was viewed as a gene set, using GeneCards^109^ for gene to pathway mapping. For each pathway we then searched for the gene sets in the OpenBEL model with largest overlap. The statistical significance of this overlap was assessed via a hyper-geometric test and corrected for multiple testing using the Benjamini-Yekutieli method under dependency^29^. In conclusion 24 KEGG/Reactome pathways could be mapped at a significance cutoff of 5% (see complete list in Supplements).

Given two mapped gene sets *A*, *B* we then calculated shortest paths between all *a* ∈ *A* and *b* ∈ *B*. The union of these shortest paths was depicted as an OpenBEL sub-graph.

## Electronic supplementary material

Supplements
