# Supplementary material for: Using Multi-Scale Genetic, Neuroimaging and Clinical Data for Predicting Alzheimer’s Disease and Reconstruction of Relevant Biological Mechanisms
Source: Sci Rep. 2018 Jul 24;8:11173. doi: 10.1038/s41598-018-29433-3 (PMC6057884; doi:10.1038/s41598-018-29433-3)
Supplement: Supplementary file 1 — Supplements [file 41598_2018_29433_MOESM1_ESM.pdf]

Using Multi-Scale Genetic, Neuroimaging and Clinical Data for Predicting Alzheimer's Disease and Reconstruction of Relevant Biological Mechanisms

Shashank Khanna, Daniel Domingo-Fernandez, Anandhi Iyappan, Mohammad Asif Emon, Martin Hofmann-Apitius, Holger Fröhlich

April 27, 2018

1 Details about ssGCCA Tuning

Tuning of the ssGCCA model implemented in "mixOmics" followed general suggestions by [Garali et al., 2017]: For each data modality (clinical, SNP, pathways, principal components) there was a separate regularization parameter $\in [0, 1]$, where 0 indicates no selection of any variable and 1 inclusion of all features. Parameters tested for clinical parameters were $\{0.5, 0.7, 1\}$, for SNPs and pathways $\{0.05, 0.1, 0.2\}$, and for principal components $\{0.25, 0.5, 0.75, 1\}$. For each parameter combination we evaluated the prediction performance of a Cox regression model via a 5-fold cross-validation, and the C-index was used as performance criterion. The number of canonical covariates was fixed to 2 after observing only minor effects on the cross-validated C-index when changing the number of canonical covariates within a small range. Note that a large number of canonical covariates would increase the chance of overfitting.

2 Supplementary Figures

Figure 1 shows a principal component plot of 926 patients. Figures 2, 3 highlight differences in the APOE4 and rs405509 status in dependency on the defined high risk and low risk groups. Figure 4 compares different prediction methods in terms of cross-validated C-index. Figure 5 compares different BN structure learning algorithm based on the cross-validated negative log-likelihood. Figures 6 - 17 depict the 12 OpenBEL networks related to specific stable edges in the BN structure.

3 Supplementary Tables

Excel sheet "SupplementaryTables.xlsx" contains:

- GBM model features with their relative influences (sheet 1)
- selection frequency of features during 10 times repeated 10-fold cross-validation (sheet 2)
- stable BN edges with >50% bootstrap frequency and >50% probability for edge direction (sheet 3)
- mapping of pathways from BN to OpenBEL sub-graphs (sheet 4)
- mapping of edges from BN to OpenBEL sub-graphs (sheet 5)
- significance of overlap of pathway pairs that are associated to stable edges in our BN (sheet 6)

4 Codes

All R-codes can be downloaded from github: `https://github.com/hfroehlich30975/AD-risk-model`.

References

[Garali et al., 2017] Garali, I., Adanyeguh, I. M., Ichou, F., Perlbarg, V., Seyer, A., Colsch, B., Moszer, I., Guillemot, V., Durr, A., Mochel, F., and Tenenhaus, A. (2017). A strategy for multimodal data integration: Application to biomarkers identification in spinocerebellar ataxia. *Briefings in Bioinformatics*.

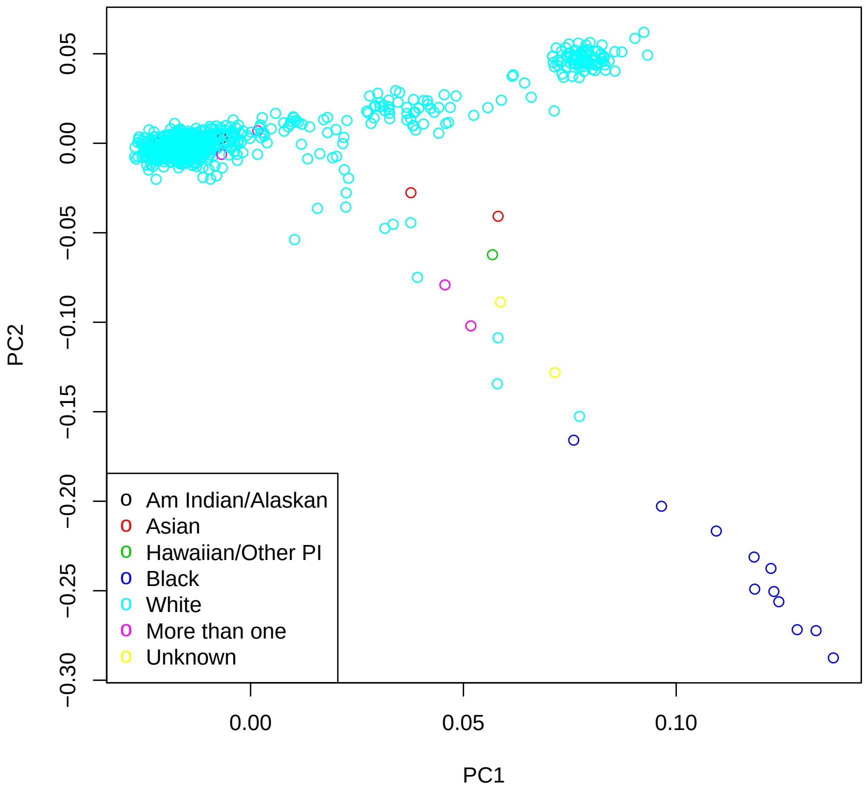

Figure 1: Principal component plot of 926 patients based on ~300,000 SNPs together with ethnicity of individuals.

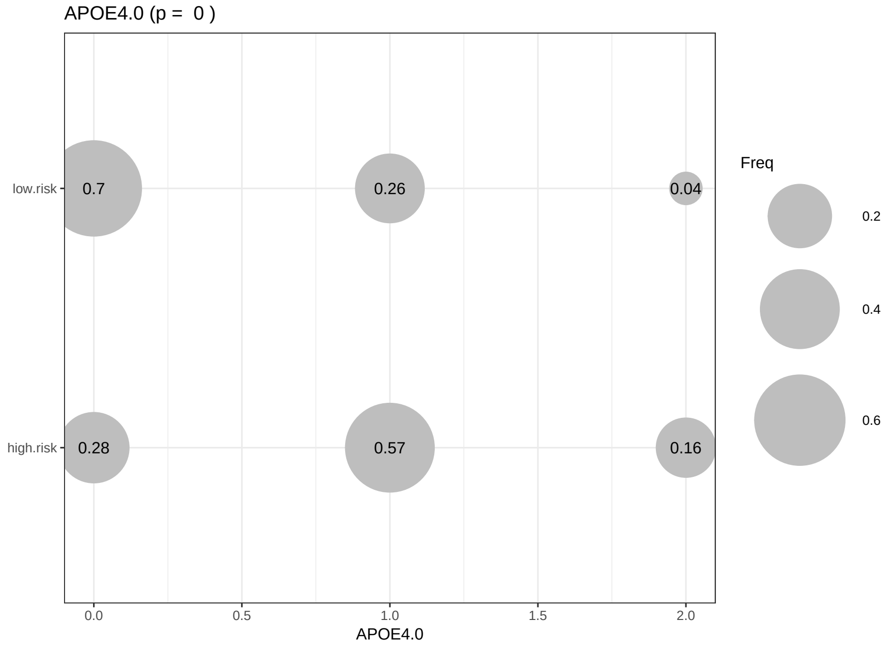

Figure 2: APOE4 status for low risk and high risk group. Coding: 0 = no $\epsilon 4$ allele, 1 = one $\epsilon 4$ allele, 2 = two $\epsilon 4$ alleles. Relative frequencies add to 1 within each patient group.

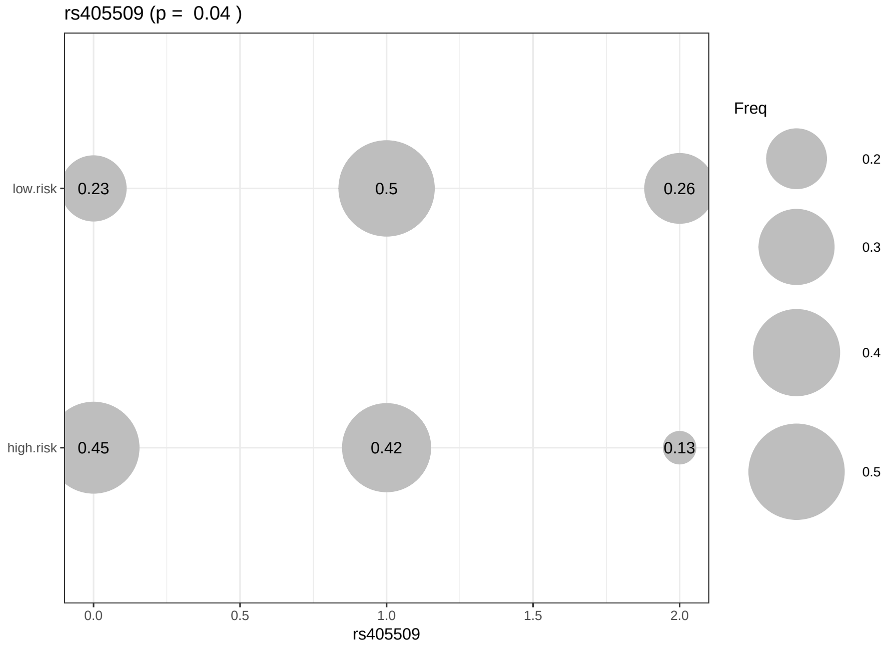

Figure 3: rs405509 status for low risk and high risk group. Allele coding: 0 = T/T, 1 = T/G, 2 = G/G. Relative frequencies add to 1 within each patient group.

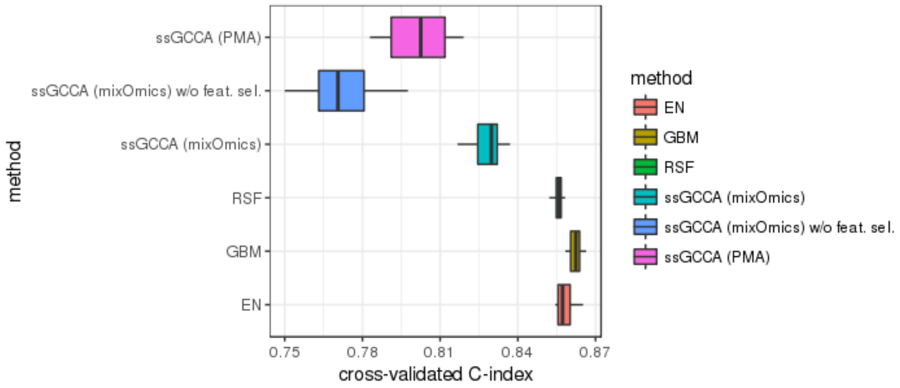

Figure 4: Comparison of different predictive methods in terms of cross-validated C-index. The boxplots show the distribution of the cross-validated C-index over 10 repeats of the cross-validation procedure.

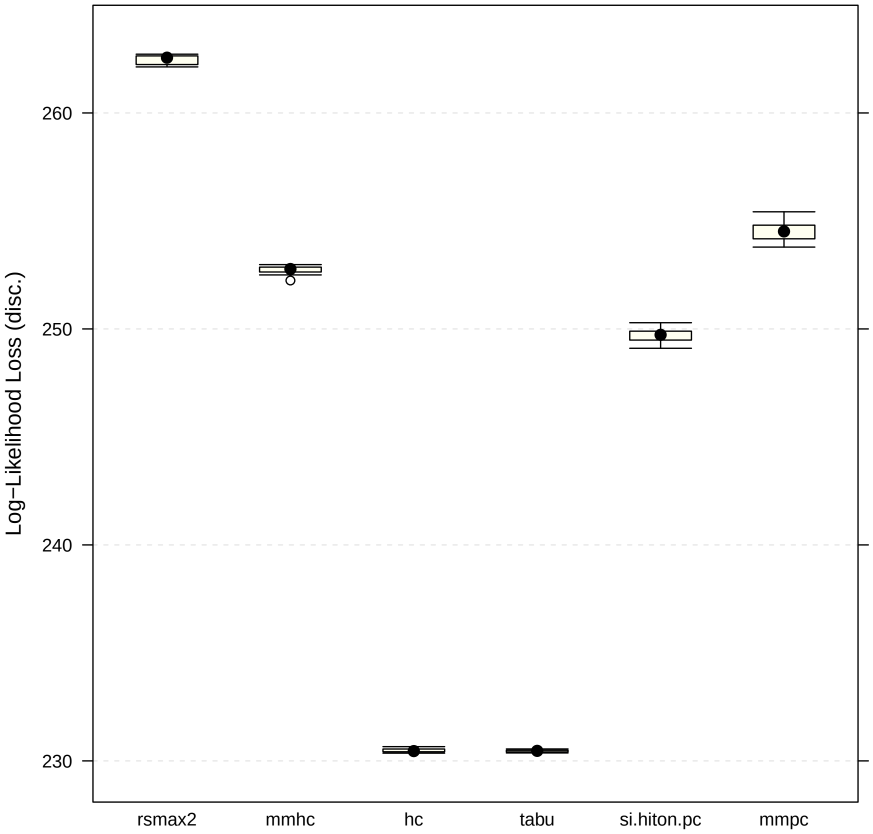

Figure 5: Cross-validated negative log-likelihood for different Bayesian Network structure learning algorithms. The lower the negative log-likelihood the better the prediction.

Adherens junction (KEGG) - Autophagy animal (KEGG)

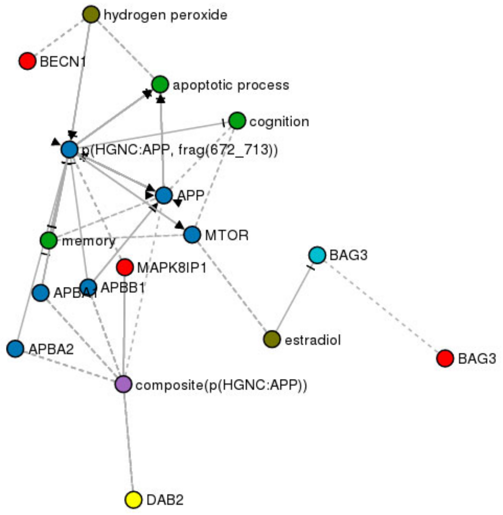

Figure 6: Mapping of stable BN edge to biological mechanisms via the OpenBEL AD graph by Kodamullil et al. (2015): Biological entities mapping to the source of the edge in the BN are drawn in yellow. Biological entities mapping to the sink of the edge are shown in red. Red edges highlight the shortest path connection between any yellow and red node. **Note**: The term "autophagy animal" was taken from KEGG, but refers to a pathway in homo sapiens.

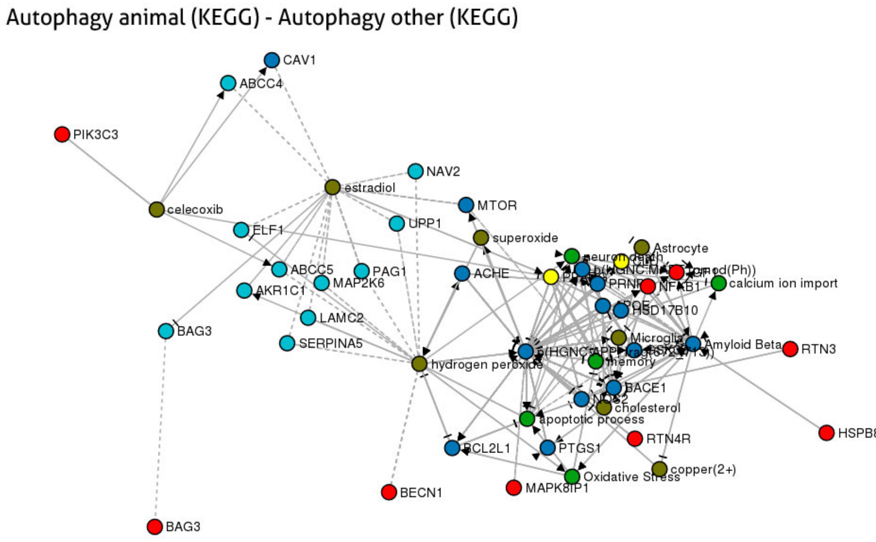

Figure 7: Mapping of stable BN edge to biological mechanisms via the OpenBEL AD graph by Kodamullil et al. (2015): Biological entities mapping to the source of the edge in the BN are drawn in yellow. Biological entities mapping to the sink of the edge are shown in red. Red edges highlight the shortest path connection between any yellow and red node. **Note**: The term "autophagy animal" was taken from KEGG, but refers to a pathway in homo sapiens.

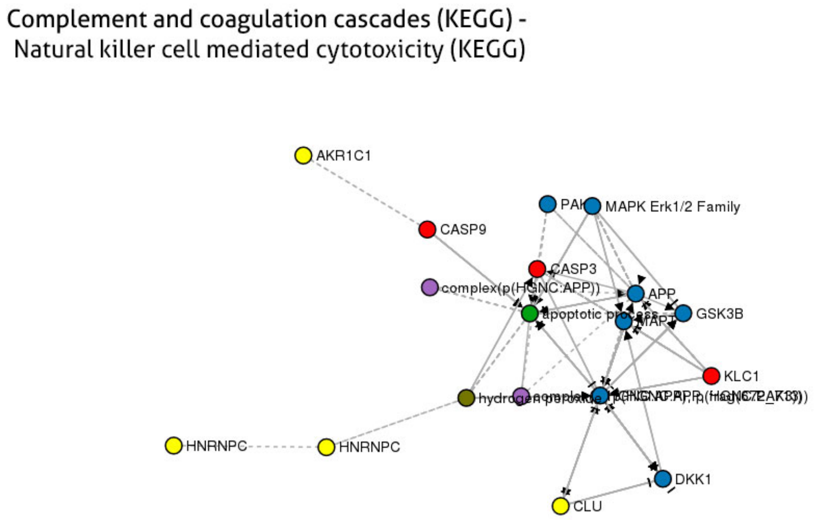

Figure 8: Mapping of stable BN edge to biological mechanisms via the OpenBEL AD graph by Kodamullil et al. (2015): Biological entities mapping to the source of the edge in the BN are drawn in yellow. Biological entities mapping to the sink of the edge are shown in red. Red edges highlight the shortest path connection between any yellow and red node.

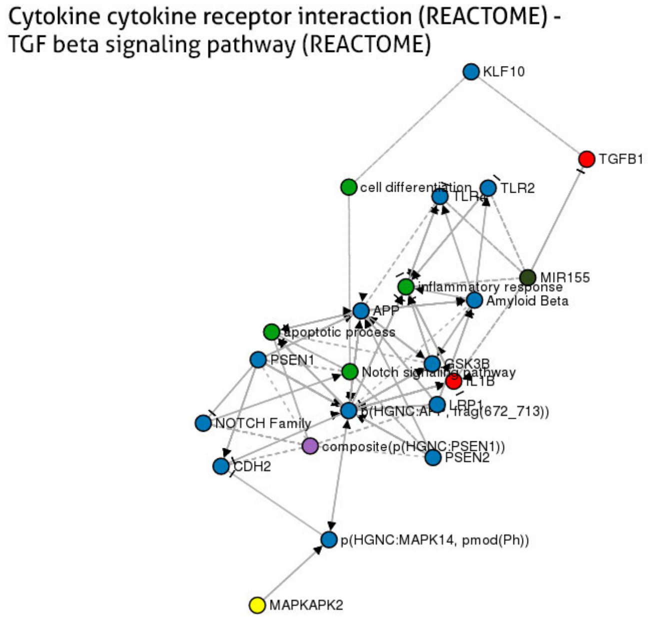

Figure 9: Mapping of stable BN edge to biological mechanisms via the OpenBEL AD graph by Kodamullil et al. (2015): Biological entities mapping to the source of the edge in the BN are drawn in yellow. Biological entities mapping to the sink of the edge are shown in red. Red edges highlight the shortest path connection between any yellow and red node.

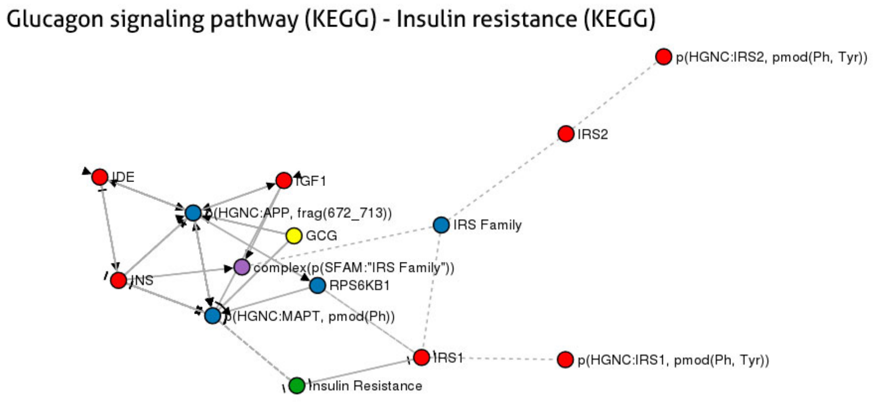

Figure 10: Mapping of stable BN edge to biological mechanisms via the OpenBEL AD graph by Kodamullil et al. (2015): Biological entities mapping to the source of the edge in the BN are drawn in yellow. Biological entities mapping to the sink of the edge are shown in red. Red edges highlight the shortest path connection between any yellow and red node.

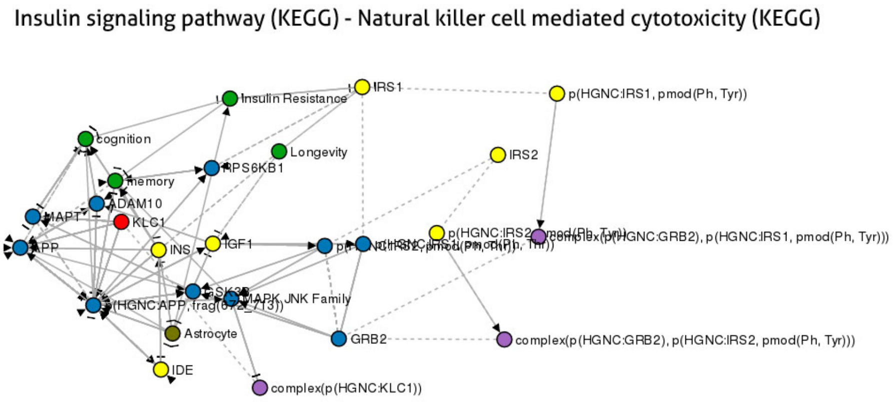

Figure 11: Mapping of stable BN edge to biological mechanisms via the OpenBEL AD graph by Kodamullil et al. (2015): Biological entities mapping to the source of the edge in the BN are drawn in yellow. Biological entities mapping to the sink of the edge are shown in red. Red edges highlight the shortest path connection between any yellow and red node.

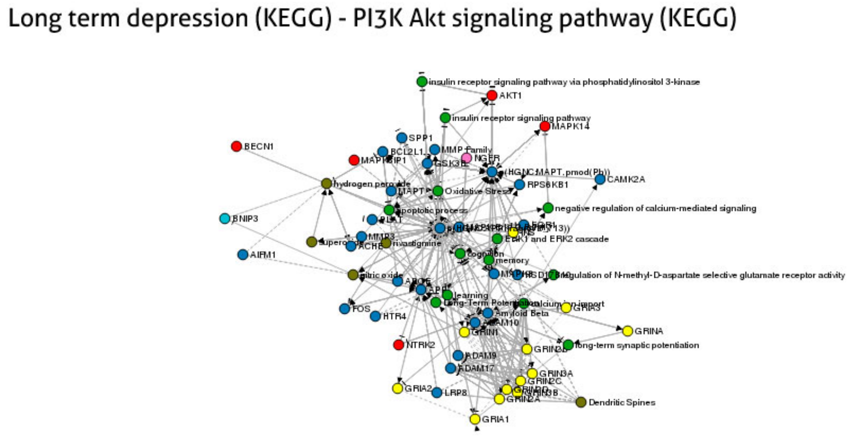

Figure 12: Mapping of stable BN edge to biological mechanisms via the OpenBEL AD graph by Kodamullil et al. (2015): Biological entities mapping to the source of the edge in the BN are drawn in yellow. Biological entities mapping to the sink of the edge are shown in red. Red edges highlight the shortest path connection between any yellow and red node.

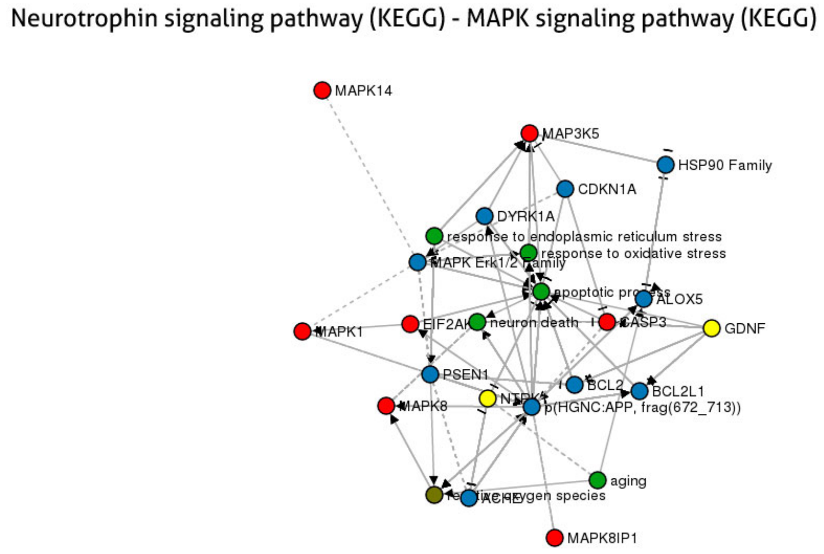

Figure 13: Mapping of stable BN edge to biological mechanisms via the OpenBEL AD graph by Kodamullil et al. (2015): Biological entities mapping to the source of the edge in the BN are drawn in yellow. Biological entities mapping to the sink of the edge are shown in red. Red edges highlight the shortest path connection between any yellow and red node.

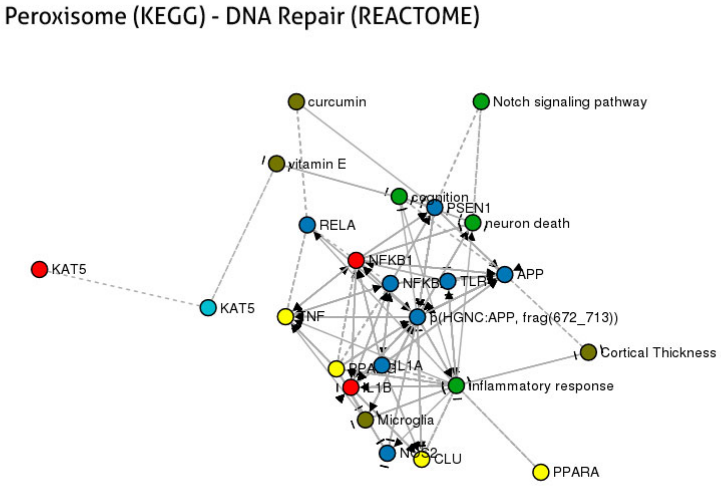

Figure 14: Mapping of stable BN edge to biological mechanisms via the OpenBEL AD graph by Kodamullil et al. (2015): Biological entities mapping to the source of the edge in the BN are drawn in yellow. Biological entities mapping to the sink of the edge are shown in red. Red edges highlight the shortest path connection between any yellow and red node.

PPAR signaling pathway (KEGG) - Glucagon signaling pathway (KEGG)

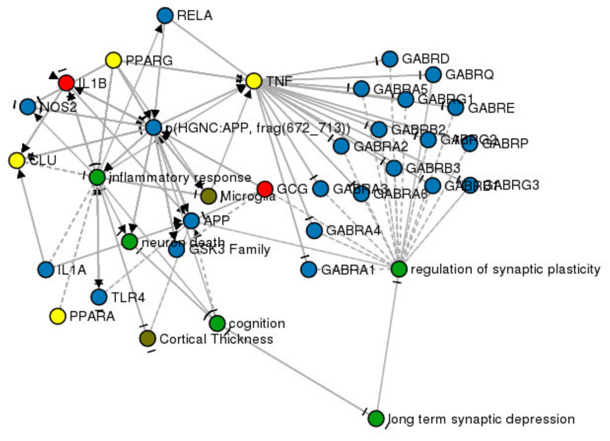

Figure 15: Mapping of stable BN edge to biological mechanisms via the OpenBEL AD graph by Kodamullil et al. (2015): Biological entities mapping to the source of the edge in the BN are drawn in yellow. Biological entities mapping to the sink of the edge are shown in red. Red edges highlight the shortest path connection between any yellow and red node.

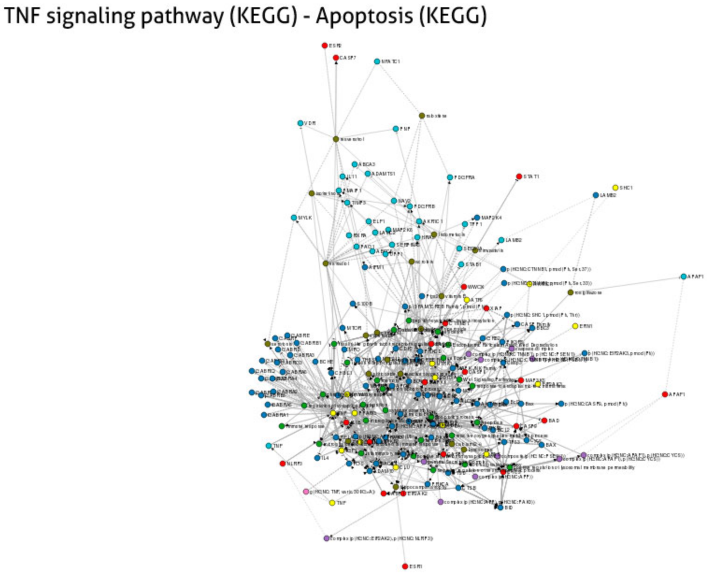

Figure 16: Mapping of stable BN edge to biological mechanisms via the OpenBEL AD graph by Kodamullil et al. (2015): Biological entities mapping to the source of the edge in the BN are drawn in yellow. Biological entities mapping to the sink of the edge are shown in red. Red edges highlight the shortest path connection between any yellow and red node.

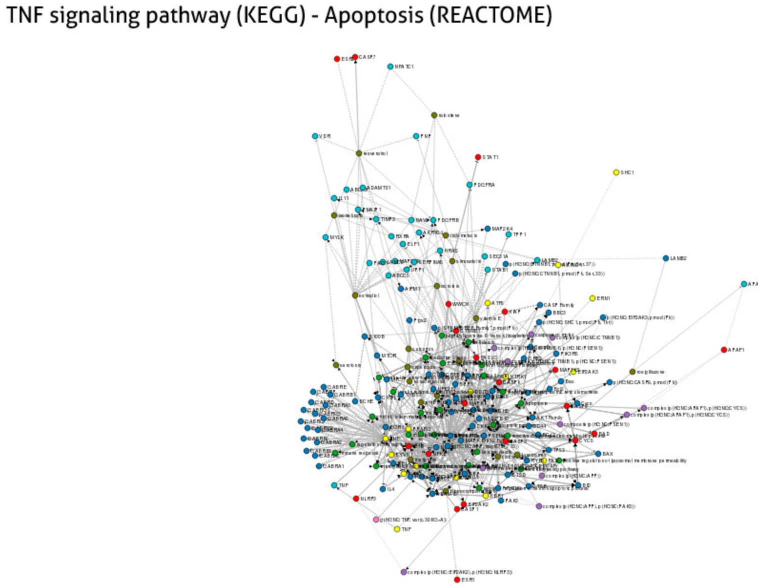

Figure 17: Mapping of stable BN edge to biological mechanisms via the OpenBEL AD graph by Kodamullil et al. (2015): Biological entities mapping to the source of the edge in the BN are drawn in yellow. Biological entities mapping to the sink of the edge are shown in red. Red edges highlight the shortest path connection between any yellow and red node.
